# The relationship between confidence and accuracy with verbal and verbal + numeric confidence scales

**DOI:** 10.1186/s41235-018-0134-3

**Published:** 2018-11-07

**Authors:** Eylul Tekin, Wenbo Lin, Henry L. Roediger

**Affiliations:** Washington University in St. Louis, One Brookings Drive, St Louis, MO 63130-4899 USA

**Keywords:** Confidence–accuracy relationship, Confidence scales, Scale ranges, Scale types, Eyewitness memory

## Abstract

Police departments often use verbal confidence measures (highly confident, somewhat confident) with a small number of values, whereas psychologists measuring the confidence–accuracy relationship typically use numeric scales with a large range of values (20-point or 100-point scales). We compared verbal and verbal + numeric confidence scales for two different lineups, using either two or four levels of confidence. We found strong confidence–accuracy relationships that were unaffected by the nature of the scale at the highest level of confidence. High confidence corresponded to high accuracy with both two- and four-level scales, and the scale type (verbal only or verbal + numeric) did not matter. Police using a simple scale of “highly confident” and “somewhat confident” can, according to our results, rest assured that high confidence indicates high accuracy on a first identification from a lineup. In addition, our two lineups differed greatly in difficulty, yet the confidence–accuracy relationship was quite strong for both lineups, although somewhat lower for the more difficult lineup.

## Significance

Police officers tend to use verbal confidence scales with only two levels of confidence on those occasions when they ask for confidence. Psychologists examining confidence–accuracy relationships usually conduct research with fine-grained numeric scales, often 20- or 100-point scales. We asked whether the same strong relation between confidence and accuracy that has been found with fine-grained scales would be replicated with two- and four-level scales with verbal statements of confidence. The answer is yes: high confidence indicates high accuracy about equally for both scale types. Further, adding numbers to verbal scales did not change performance. We used two different lineups that varied markedly in difficulty. Although correct identification rates were low for the difficult set, high confidence responses still provided highly accurate responding at 87%. We conclude that high confidence responses on verbal scales typical of those used by police provide highly accurate responses.

### The relationship between confidence and accuracy with verbal and numeric confidence scales

The relationship between confidence and accuracy has long been debated within eyewitness research, but recently a resolution to the debate has occurred. Studies from the 1980s until relatively recently, using a point-biserial correlation technique (a correlation between the dichotomous accuracy variable and the corresponding confidence ratings), led to the conclusion that there was little to no relationship between confidence and accuracy (Kassin, Ellsworth, & Smith, 1989; Wells & Murray, 1984). However, Juslin, Olsson, and Winman (1996) argued that even when the point-biserial correlation was small, a strong confidence–accuracy (CA) relationship could still exist in the data. They computed a calibration plot using the formula *C = # correct ID/(# correct ID + # incorrect ID)* that includes all filler IDs from target-absent (TA) and target-present (TP) lineups and showed a strong CA relation even when the point-biserial correlation was low. Because fillers in TP lineups are known to be innocent, another way to compute calibration plots is to exclude these filler IDs. This approach, now called confidence–accuracy calibration characteristic plots (CAC; Mickes, 2015), provide measures of confidence (in bins) on the abscissa and measures of accuracy from low to high on the ordinate. Typically, these plots show that high confidence is associated with quite high accuracy (Mickes, 2015; Palmer, Brewer, Weber, & Nagesh, 2013; Sauer, Brewer, Zweck, & Weber, 2010; Wixted, Mickes, Clark, Gronlund, & Roediger, 2015; for a review, see Wixted & Wells, 2017). This outcome seems true of initial witness reports. If a witness has been repeatedly tested and has moved from a low confidence initial identification to a high confidence courtroom identification, then an error is likely. High confidence in the courtroom usually should not be weighed heavily (if at all) if the witness’s confidence at the first lineup was low.

Studies using CAC analysis have often employed numeric confidence scales (e.g. 20-point scales or 100-point scales), which are not reflective of eyewitness identification as conducted by police departments. Unlike laboratory studies, police departments usually have eyewitnesses verbally express their confidence or use a small range of confidence scales (perhaps highly confident or somewhat confident) instead of 100-point scales. Behrman and Richards (2005) reported that eyewitnesses typically used phrases such as “He resembles the guy” or “I think he did it” to indicate their certainty (or lack thereof). Wells (2014) reported that the Houston Police Department used a three-level verbal scale (positive, strong tentative, or weak tentative) for eyewitness identifications. Together, these police procedures raise an important question: Are such verbal confidence scales with few levels of confidence as predictive of eyewitness accuracy as the more fine-grained numeric scales used in laboratory studies? This is the issue we addressed in our paper, but we first review related findings.

Dodson and Dobolyi (2015a) showed that providing verbal or numeric labels and varying the number of confidence points on a 100-point scale (6 points: 0, 20, 40, 60, 80, 100; or 11 points: 0, 10, 20, 30, 40, 50, 60, 70, 80, 90, 100) did not change the CA relationship for eyewitness identification. Nonetheless, they employed a 100-point scale, which is unlikely to be used by police departments. Recently, Tekin and Roediger (2017) compared narrow ranges (4- and 5-point scales) to wider ranges (20- and 100-point scales) and concluded that the scale range did not affect the CA relationship with numeric scales. However, they used unrelated words and faces as materials, not lineups.

In the present study, we directly compared two- and four-level scales using lineups, because the four-level scale can be directly compared to the two-level scale by combining levels. The four-level scale may also be applicable in some police departments. Although previous research from our lab revealed that the 4-point and wider (e.g. 20-point, 100-point) scales did not differ from one another in the CA relation (Tekin & Roediger, 2017), no one has examined the issue with smaller scales (e.g. two- and four-level scales). Importantly, we used verbal confidence statements, as often used by police departments, and we also examined whether providing numerical values for the verbal confidence statements provided any benefits to the CA relationship compared to only verbal statements. In a quest for external validity, we did not compare verbal scales to just numeric scales because eyewitnesses are unlikely to give a numeric confidence without a verbal statement; thus, we added numbers to the verbal confidence scales to make them comparable. In addition, we employed two different material sets to establish some generalizability. Interestingly, one lineup turned out to be much more difficult for individuals than the other lineup and thus we can examine the effect of lineup difficulty on the CA relationship.

The current experiment addressed three primary questions. First, do small scales produce similar CA relationships (e.g. 2 values of confidence compared to 4 values), as is true for larger numeric scales (e.g. 20 points compared to 100 points)? Second, does adding numbers to purely verbal scales affect the CA relationship compared to using only verbal scales? Third, do the results replicate across two different sets of material (crime scenes and associated lineups)? To answer these questions, individuals viewed two videos, made identifications for possible suspects in each video with TP and TA lineups, and then indicated their confidence on either: (1) a verbal-only two-level scale; (2) a verbal + numeric 2-point scale; (3) a verbal-only four-level scale; or (4) a verbal + numeric 4-point scale.

## Method

### Participants

To detect a small effect using chi-square tests, an a priori power analysis was conducted using a small effect size (*φ* = 0.1), an alpha of 0.05, *df* of 1, and a power of 0.80. Based on this, a sufficient number of observations was 785. We recruited 833 Amazon’s Mechanical Turk (MTurk) workers (mean age = 34.25, *SD* = 10.04) who were located in the United States and had high completion and performance rates (> 90%) in prior studies. Seven individuals were eliminated based on the questionnaire at the end of the experiment (four did not view the videos, three did not follow the instructions) and one due to experiment error. Of the remaining 825 individuals, 22 of them provided one observation and the rest provided two observations (one per lineup), totaling up to 1628 data points.^1^

### Design and material

The experiment employed a 2 × 2 × 2 mixed design. The scale range (two levels vs four levels) and the scale type (verbal-only vs verbal + numeric) were manipulated between-subjects, whereas the lineup type (TP vs TA) was manipulated within-subjects. Individuals viewed two videos and were later tested with a TP lineup for one of the videos and a TA lineup for the other. The order of the videos and the lineups were counterbalanced. Thus, every 16 participants constituted a complete experimental counterbalancing.

Two silent mock crime videos (Set A and Set B), in which a young, white male stole a laptop, were used as materials (Mickes, Flowe, & Wixted, 2012; L. Mickes, personal communication, September 12, 2017). Both videos were approximately 30 s long and the suspects showed their faces transiently to the camera for about 3–4 s in both videos.

**Fig. 1:**
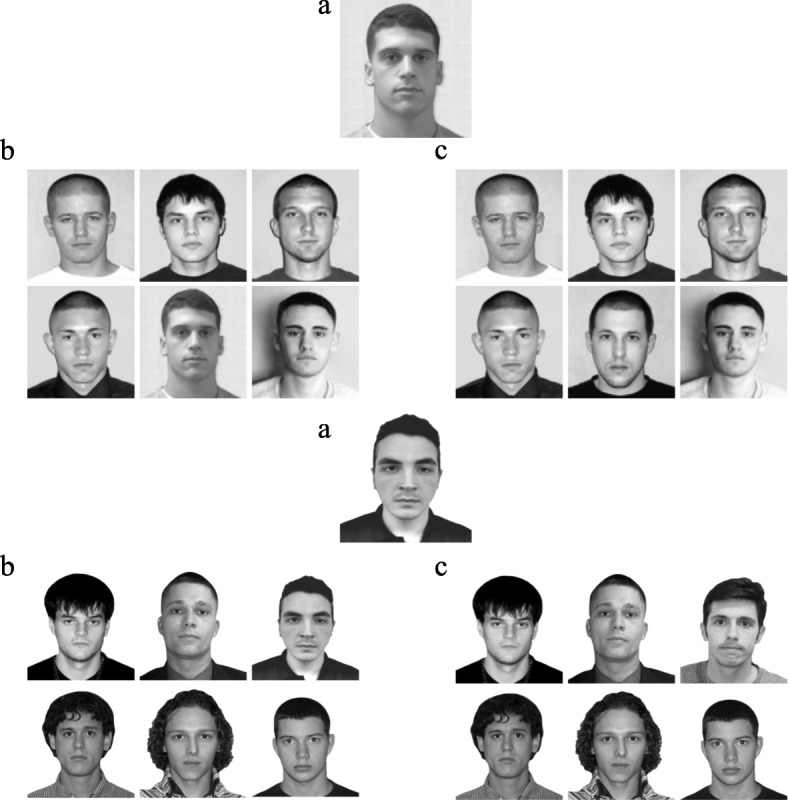
Set A (*top*) and Set B (*bottom*): (**a**) represents the suspects and (**b**) and (**c**) are the TP and TA lineups, respectively

TP and TA lineups for each video consisted of six black and white headshots and they were presented simultaneously in a matrix of two rows with three faces in each row. The positions of the headshots were randomized across subjects. The TP lineups included five fillers and the suspect whereas the TA lineups were composed of six fillers (see Fig. 1). The fillers were selected based on their resemblance (e.g. hair color, age) to the suspects. The TA lineup only included fillers.

The distractor tasks between the video and test phases of the experiment were questionnaires for openness to experience and conscientiousness that consisted of 20 short statements each (Costa & McCrae, 1992). The order of the questionnaires was counterbalanced across individuals.

### Procedure

Participants were randomly assigned to one of the four between-subject conditions and were instructed that they would see videos and then later complete memory tests about the videos. After viewing the first video, they completed one of the personality questionnaires. Then they were given instructions about the lineup they were about to see and were asked to pick a suspect from the lineup, if he were there. Participants were informed that the suspect may or may not be present in the lineup. If the suspect was not in the lineup, they were told to select the “Not Present” option. They were then presented with either a TP or a TA lineup and were asked to make an identification decision. Immediately following their decision, they were asked to indicate their level of confidence on a confidence scale in accord with their assigned condition. Individuals who were assigned to the verbal-only two-level scale were presented with options of “not sure at all” and “absolutely sure” following their identification decision, whereas those who were assigned to the verbal-only four-level scale were presented with options of “not sure at all,” “somewhat sure,” “very sure,” and “absolutely sure.” For participants who were in the verbal + numeric condition, the labels also had a corresponding number next to them (e.g. “3-very sure”). Following their confidence judgments, individuals were asked to recall U.S. states for 1 min before proceeding to the second video, just to provide mental separation between the two parts of the experiment.

The second half of the experiment was identical to the first half except for the difference in the material, questionnaire, and lineup. Individuals viewed the second mock crime video and took the alternate personality questionnaire. Finally, they were given the same lineup instructions and were presented with either a TP or a TA lineup that was the same as the first type of lineup. They rated their confidence on the same scale as used for the first lineup, but if they had initially seen a TP lineup, they received a TA lineup the second time (and vice versa). The experiment lasted approximately 8 min.

## Results

Table 1 shows frequencies of different identification responses (correct or suspect ID; incorrect [filler ID]; or not present or non-ID) separately for TP and TA lineups and material sets. The first point to note is that the two sets of material differed greatly in correct performance, with *d’* scores of 1.98 and 0.88 and suspect identification rates of 73% and 27% for Set A and Set B, respectively. In fact, when faced with a TP lineup for Set B materials, the most common response was to reject the lineup by saying the suspect did not appear in it (41% of the responses, relative to only 15% for Set A). This large difference in difficulty is a benefit in addressing the issues in our paper, because we can see if our findings concerning the CA relationship are the same with easy and difficult lineups.

**Table 1:** Frequency and percentage of identification responses in each material set for target-present (TP) and target-absent (TA) lineups

	Identification response: TP lineup						
	Suspect ID (Correct)		Filler ID (Incorrect)		Non-ID (Not present)		Total
Material	n	%	n	%	n	%	n
Set A	297	73.0	50	12.3	60	14.7	407
Set B	109	26.8	132	32.5	165	40.6	406
Overall	406	49.9	182	22.4	225	27.7	813
	Identification response: TA lineup						
	Non-ID (Correct rejection)		Filler ID (False identification)		Total		
Material	n	%	n	%	n		
Set A	198	48.9	207	51.1	405		
Set B	244	59.5	166	40.5	410		
Overall	442	54.2	373	45.8	815		

n stands for number of observations

To analyze the data in Table 1, we first tested the material effects. For TP lineups, a 3 (identification response) × 2 (materials) chi-square analysis revealed a significant materials effect, χ^2^ (2, *N* = 813) = 173.00, *p* < 0.001, indicating that Set A had higher suspect identifications and lower lure identifications and false rejections. Similarly, for TA lineups, a 2 (identification response) × 2 (materials) chi-square analysis revealed a significant material effect, χ^2^ (1, *N* = 815) = 8.84, *p* = 0.003, indicating that Set A had higher false identifications and lower correct rejections compared to Set B. Again, this outcome serves our purpose for we can ask if the different ranges of scales (two- and four-level) and the verbal and verbal + numeric scales show similar effects for easy and difficult materials.

Given these results, further analyses were conducted for both Set A and Set B separately, as well as aggregating the results across the two sets. We report the separate analyses only when their results differed from each other or from the aggregated results. To assess the effects of scale range and scale type, we first conducted chi-square tests to compare frequencies of identification responses for TP and TA lineups and then CAC plots to compare accuracy levels across different confidence levels. Here, we only report the results from CAC plots because they are the primary interest. The results from chi-square tests resembled those of CAC plots and are available, with our data, in the Open Science Framework, osf.io/xu7g5.

### CAC analyses

**Fig. 2:**
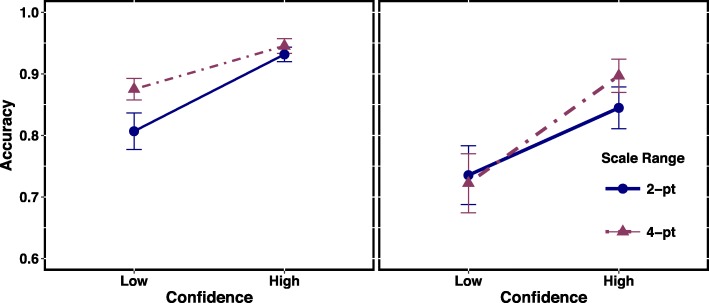
Comparison of the 2-point scale to the 4-point scale for Set A (*left*) and Set B (*right*). *Error bars* indicate standard errors

**Fig. 3:**
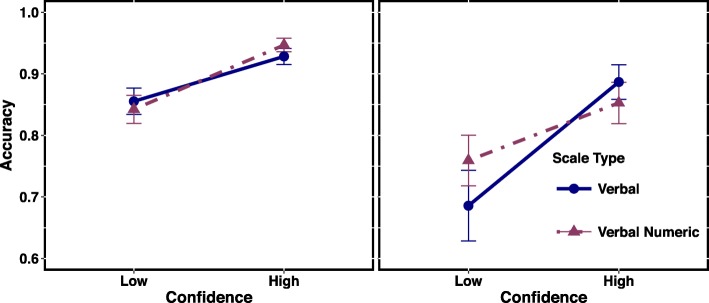
Comparison of the verbal scale to the verbal and numeric scale for Set A (*left*) and Set B (*right*). *Error bars* indicate standard errors

Figures 2 and 3 show CAC plots for scale ranges and scale types, respectively, for Set A and Set B sets separately. For CAC plots, we binned the lowest two ratings of the 4-point scale to compare them to the lowest rating of the 2-point scale (low confidence) and the highest two ratings of the 4-point scale to compare them to the highest rating of the 2-point scale (high confidence). Table 2 provides the frequencies of responses at each confidence level. For low and high confidence responses, accuracy (A) was computed using the formula A = # correct suspect IDs/(# correct suspect IDs + # incorrect suspect IDs/6), as recommended by Mickes (2015). First, replicating prior work, when people make a high confident decision (even on a 2-point scale), they are highly accurate; they were 94% correct for the Set A lineup and over 87% correct for Set B lineup.

**Table 2:** Frequencies of identification responses at each confidence level for target-present (TP) and target-absent (TA) lineups

Identification response: TP lineup				
	Confidence level			
4 points	1	2	3	4
Suspect ID	10	100	80	21
Filler ID	12	51	12	3
Non-ID	12	48	42	18
Identification response: TA lineup				
	Confidence level			
	1	2	3	4
Filler ID	22	110	37	8
Non-ID	16	100	78	38
Identification response: TP lineup				
	Confidence level			
2 points		1	2	
Suspect ID		73	122	
Filler ID		70	34	
Non-ID		37	68	
Identification response: TA lineup				
	Confidence level			
		1	2	
Filler ID		123	73	
Non-ID		68	142	

#### Scale range

For Set A (Fig. 2, left), low confidence responses on the 4-point scale led to 7% greater accuracy relative to the 2-point scale, whereas the difference was only 1% for high confidence responses and the standard error bars overlapped. For Set B (Fig. 2, right), standard error bars overlapped for both confidence levels, indicating that accuracy levels for the two scale ranges did not differ for low and high confidence responses. Even though the results for Set A and Set B differed for low confidence responses, both sets showed high accuracy for high confidence responses (albeit 7% higher for Set A than B). Furthermore, comparison of the highest points on the scales (i.e. only 4 points on the 4-point scale and 2 points on the 2-point scale) did not change the results. Accuracy for confidence ratings of 4 was 0.94. For more details, see supplementary analyses.

#### Scale type

For both Set A (Fig. 3, left) and Set B (Fig. 3, right), accuracy levels for the verbal and verbal + numeric scales did not differ across confidence levels. For Set A, the verbal and verbal + numeric scales led to almost identical accuracy. Even though they differed slightly for low confidence responses of Set B, the standard error bars overlapped. Adding numbers to verbal statements of confidence did not affect accuracy. Bayes factor analyses for the claimed null effects in this section and the previous section appear in the supplementary analyses.

#### Material set

**Fig. 4:**
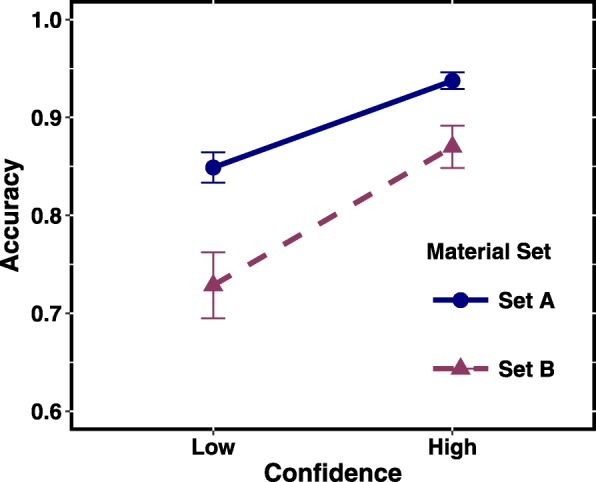
Comparison of the easy (Set A) and difficult (Set B) materials. *Error bars* indicate standard errors

We also tested whether the difficulty of material sets affected accuracy at different confidence levels. Figure 4 shows the CAC plots for the two material sets. For both low and high confidence, Set A led to higher accuracy levels than Set B (a 7% difference at the high level of confidence, with non-overlapping error bars). Thus, unlike scale range and type, material difficulty did affect accuracy levels even for high confidence responses. Still, even for the quite difficult Set B materials, high confidence indicated relatively high accuracy (87%).

## General discussion

Police departments in the U.S. rarely have specific guidelines about the range and type of confidence scales to use in eyewitness identification, but they often use verbal scales with only two or three levels of confidence. The main purpose of the current experiment was to investigate whether the range and type of confidence scales used by police departments led to similar CA relationship as the numeric scales in laboratory studies.

The two-level confidence scale in our experiment consisted of two extreme confidence statements (e.g. “Absolutely sure” and “Not sure at all”), whereas the four-level scales provided intermediate values. Even though it seems intuitive to assume that high confidence on a four-level scale would lead to better accuracy than high confidence on a two-level scale, the results demonstrated otherwise. For high confidence responses, both 2-point and 4-point scales yielded similar accuracy levels, although some differences were noted at lower levels of the scale. Tekin and Roediger (2017) reported the same outcome for 4-, 5-, 20-, and 100-point confidence scales. High confidence indicated high accuracy; a 4 on a 4-point scale produces roughly the same level of accuracy as 100 on a 100-point scale.

The question that matters for judges and jurors is whether accuracy of the eyewitness would change if confidence judgments were made more or less fine-grained scales. The answer is conclusively *no*. When eyewitnesses are highly confident, they are highly accurate regardless of how many levels of confidence the scale provides. This conclusion is in line with recent evidence demonstrating that variables such as distance from the perpetrator during the crime do not affect accuracy of identifications made with high confidence (Semmler, Dunn, Mickes, & Wixted, 2018). Scale range is yet another variable that has no effect on high confidence eyewitness accuracy. This is especially surprising given that the range of a confidence scale would seem to change how “high confidence” is defined, but it is a comforting outcome for the legal system.

The second issue examined whether providing numeric labels to verbal scales improved the CA relationship compared to verbal only scales. The answer is no; for both low and high confidence responses, the CAC plots for these scale types led to similar accuracy. Dodson and Dobolyi (2015b, 2017), however, have reported that verbal confidence statements, especially ones accompanied by a justification, increased variability in observers’ (e.g. police or jurors) interpretation of those confidence statements. Thus, even though accuracy of the eyewitness was not subject to change with the additional numeric labels, providing numbers might reduce ambiguity in observers’ interpretation.

We used two material sets that differed greatly in difficulty. Nonetheless, both sets of material provided similar patterns of results for the two scale ranges and scale types: When the identification was made with high confidence, the nature of the scale did not affect accuracy. However, difficulty of the materials did affect accuracy. Nevertheless, even for a difficult lineup, high confidence judgments were associated with high accuracy. Our results point to the need for a wide array of stimulus materials for eyewitness experiments to be developed and placed in an open repository to ensure that results generalize across lineup types.

In conclusion, replicating and expanding on previous findings (Dodson & Dobolyi, 2015a; Tekin & Roediger, 2017), we demonstrated that the verbal scales with relatively few values used by police departments lead to CA relationships similar to those of more fine-grained scales. Providing numeric labels for verbal scales did not improve the CA relationship. For the various scale types, high confidence indicates high accuracy (around 90% combined over the two material sets in our studies). This figure is somewhat lower than in other studies, but most of those used materials producing higher rates of accuracy than our Set B materials.

## Abbreviations

CA: Confidence–accuracy
CAC: Confidence–accuracy calibration
TA: Target-absent
TP: Target-present

## Footnotes

1 When the experiment was initially designed, individuals were able to select both a suspect and “Not Present” option; some of the participants selected both. These individuals were dropped from the data analysis. This problem was fixed before testing the bulk of the participants.
